# Learning causal networks from systems biology time course data: an effective model selection procedure for the vector autoregressive process

**DOI:** 10.1186/1471-2105-8-S2-S3

**Published:** 2007-05-03

**Authors:** Rainer Opgen-Rhein, Korbinian Strimmer

**Affiliations:** 1 Department of Statistics, Ludwig-Maximilians-Universität München, Ludwigstraße 33, D-80539 München, Germany; 2 Institute for Medical Informatics, Statistics and Epidemiology (IMISE), University of Leipzig, Härtelstr. 16-18, 04107 Leipzig, Germany

## Abstract

**Background:**

Causal networks based on the vector autoregressive (VAR) process are a promising statistical tool for modeling regulatory interactions in a cell. However, learning these networks is challenging due to the low sample size and high dimensionality of genomic data.

**Results:**

We present a novel and highly efficient approach to estimate a VAR network. This proceeds in two steps: (i) improved estimation of VAR regression coefficients using an analytic shrinkage approach, and (ii) subsequent model selection by testing the associated partial correlations. In simulations this approach outperformed for small sample size all other considered approaches in terms of true discovery rate (number of correctly identified edges relative to the significant edges). Moreover, the analysis of expression time series data from *Arabidopsis thaliana *resulted in a biologically sensible network.

**Conclusion:**

Statistical learning of large-scale VAR causal models can be done efficiently by the proposed procedure, even in the difficult data situations prevalent in genomics and proteomics.

**Availability:**

The method is implemented in R code that is available from the authors on request.

## Background

The vector autoregressive regression (VAR) model is an approach to describe the interaction of variables through time in a complex multivariate system. It is very popular in economics [1] but with few exceptions [2] it has not been widely used in systems biology, where it could be employed to model genetic networks or metabolic interactions. One possible reason for this is that while the statistical properties of the VAR model are well explored [3], its estimation from sparse data and subsequent model selection is very challenging due to the large number of parameters involved [4].

In this paper we develop a procedure for effectively learning the VAR model from small sample genomic data. In particular, we describe a novel model selection procedure for learning causal VAR networks from time course data with only a few time points, and no or little replication. This procedure is based on regularized estimation of VAR coefficients, followed by subsequent simultaneous significance testing of the corresponding partial correlation coefficients.

Once the VAR model has been learned from the data, it allows to elucidate possible underlying causal mechanisms by inspecting the Granger causality graph implied by the non-zero VAR coefficients.

The remainder of the paper is organized as follows. In the next section we first give the definition of a vector autoregressive process and recall the standard estimation. Subsequently, we describe our approach to regularized inference and to network model selection. This is followed by computer simulations comparing a variety of alternative approaches. Finally, we analyze data from an *Arabidopsis thaliana *expression time course experiment.

## Methods

### Vector autoregressive model

We consider vector-valued time series data ***x***(*t*) = (*x*_1_(*t*),...,*x*_*p*_(*t*)). Each component of this row vector corresponds to a variable of interest, e.g., the expression level of a specific gene or the concentration of some metabolite in dependence of time. The vector autoregressive model specifies that the value of ***x***(*t*) is a linear combination of those of earlier time points, plus noise,

x(t)=c+∑i=1mx(t−iL)Bi+εi.     (1)
 MathType@MTEF@5@5@+=feaafiart1ev1aaatCvAUfKttLearuWrP9MDH5MBPbIqV92AaeXatLxBI9gBaebbnrfifHhDYfgasaacH8akY=wiFfYdH8Gipec8Eeeu0xXdbba9frFj0=OqFfea0dXdd9vqai=hGuQ8kuc9pgc9s8qqaq=dirpe0xb9q8qiLsFr0=vr0=vr0dc8meaabaqaciaacaGaaeqabaqabeGadaaakeaaieWacqWF4baEcqGGOaakcqWG0baDcqGGPaqkcqGH9aqpcqWFJbWycqGHRaWkdaaeWbqaaiab=Hha4jabcIcaOiabdsha0jabgkHiTiabdMgaPjabdYeamjabcMcaPiab=jeacnaaBaaaleaacqWGPbqAaeqaaOGaey4kaScccmGae4xTdu2aaSbaaSqaaiabdMgaPbqabaGccqGGUaGlcaWLjaGaaCzcaiabcIcaOiabigdaXiabcMcaPaWcbaGaemyAaKMaeyypa0JaeGymaedabaGaemyBa0ganiabggHiLdaaaa@4EFF@

In this formula *m *is the order of the VAR process, *L *the time lag, and ***c ***a 1 × *p *vector of means. The errors ***∈***_*i *_are assumed to have zero mean and a *p *× *p *positive definite covariance matrix **Σ**. The matrices ***B***_*i *_with dimension *p *× *p *represent the dynamical structure and thus contain the information relevant for reading off the causal relationships.

The autoregressive model has the form of a standard regression problem. Therefore, estimation of the matrices ***B***_*i *_is straightforward. A special case considered in this paper is when both *m *and *L *are set to 1. Then the above equation reduces to the VAR(1) process

***x***(*t *+ 1) = ***c ***+ ***x***(*t*)***B ***+ ***ε***.     (2)

We now denote the centered *matrices of observations *corresponding to ***x***(*t *+ 1) and ***x***(*t*) by ***X***_*f *_("future") and ***X***_*p *_("past"), respectively, i.e. Xp=[x(1)…x(n−1)]
 MathType@MTEF@5@5@+=feaafiart1ev1aaatCvAUfKttLearuWrP9MDH5MBPbIqV92AaeXatLxBI9gBaebbnrfifHhDYfgasaacH8akY=wiFfYdH8Gipec8Eeeu0xXdbba9frFj0=OqFfea0dXdd9vqai=hGuQ8kuc9pgc9s8qqaq=dirpe0xb9q8qiLsFr0=vr0=vr0dc8meaabaqaciaacaGaaeqabaqabeGadaaakeaaieWacqWFybawdaWgaaWcbaGaemiCaahabeaakiabg2da9maadmaabaqbaeqabmqaaaqaaiab=Hha4jabcIcaOiabigdaXiabcMcaPaqaaiablAcilbqaaiab=Hha4jabcIcaOiabd6gaUjabgkHiTiabigdaXiabcMcaPaaaaiaawUfacaGLDbaaaaa@3E35@ and Xf=[x(2)…x(n)]
 MathType@MTEF@5@5@+=feaafiart1ev1aaatCvAUfKttLearuWrP9MDH5MBPbIqV92AaeXatLxBI9gBaebbnrfifHhDYfgasaacH8akY=wiFfYdH8Gipec8Eeeu0xXdbba9frFj0=OqFfea0dXdd9vqai=hGuQ8kuc9pgc9s8qqaq=dirpe0xb9q8qiLsFr0=vr0=vr0dc8meaabaqaciaacaGaaeqabaqabeGadaaakeaaieWacqWFybawdaWgaaWcbaGaemOzaygabeaakiabg2da9maadmaabaqbaeqabmqaaaqaaiab=Hha4jabcIcaOiabikdaYiabcMcaPaqaaiablAcilbqaaiab=Hha4jabcIcaOiabd6gaUjabcMcaPaaaaiaawUfacaGLDbaaaaa@3C46@. In this notation the ordinary least squares (OLS) estimate can be written as

B^
 MathType@MTEF@5@5@+=feaafiart1ev1aaatCvAUfKttLearuWrP9MDH5MBPbIqV92AaeXatLxBI9gBaebbnrfifHhDYfgasaacH8akY=wiFfYdH8Gipec8Eeeu0xXdbba9frFj0=OqFfea0dXdd9vqai=hGuQ8kuc9pgc9s8qqaq=dirpe0xb9q8qiLsFr0=vr0=vr0dc8meaabaqaciaacaGaaeqabaqabeGadaaakeaaieWacuWFcbGqgaqcaaaa@2DD1@^OLS ^= (***X***_*p *_^*T *^***X***_*p*_)^-1 ^***X***_*p *_^*T *^***X***_*f*_.     (3)

This is also the maximum likelihood (ML) estimate assuming the normal distribution. The coefficients of higher-order VAR models may be obtained in a corresponding fashion [3].

### Small sample estimation using James-Stein-type shrinkage

Genomic time course data contain only few time points (typically around *n *= 10) and often little or no replication – hence the above restriction on VAR(1) models with unit lag. Furthermore, it is known that for small sample size the least squares and maximum likelihood methods lead to statistically inefficient estimators. Therefore, application of the VAR model to genomics data requires some form of regularization. For instance, a full Bayesian approach could be used. However, for the VAR model the choice of a suitable prior is difficult [4].

Here, as a both computationally and statistically efficient alternative, we propose to apply James-Stein-type shrinkage, a method related to empirical Bayes [5,6]. This procedure has the advantage that it is computationally as simple as OLS, yet still produces efficient estimates for small samples.

Following [6,7] we now review how an unconstrained covariance matrix may be estimated using shrinkage. In the next section we then show how this result may be used to obtain shrinkage estimates of VAR coefficients.

Assuming centered data ***X ***for *p *variables (columns) the unbiased empirical estimator of the covariance matrix is S=1n−1XTX
 MathType@MTEF@5@5@+=feaafiart1ev1aaatCvAUfKttLearuWrP9MDH5MBPbIqV92AaeXatLxBI9gBaebbnrfifHhDYfgasaacH8akY=wiFfYdH8Gipec8Eeeu0xXdbba9frFj0=OqFfea0dXdd9vqai=hGuQ8kuc9pgc9s8qqaq=dirpe0xb9q8qiLsFr0=vr0=vr0dc8meaabaqaciaacaGaaeqabaqabeGadaaakeaaieWacqWFtbWucqGH9aqpcaWLa8+aaSaaaeaacqaIXaqmaeaacqWGUbGBcqGHsislcqaIXaqmaaGae8hwaG1aaWbaaSqabeaacqWGubavaaGccqWFybawaaa@3885@. For small number of observations ***S ***is known to be inefficient and also ill-conditioned (singular!) for *n *<*p*. A more efficient estimator may be furnished by shrinking the empirical correlations *r*_*ij *_towards zero and the empirical variances *v*_*i *_against their median. This leads to the following expressions for the components of a shrinkage estimate S*:

skl∗=rkl∗vk∗vl∗     (4)
 MathType@MTEF@5@5@+=feaafiart1ev1aaatCvAUfKttLearuWrP9MDH5MBPbIqV92AaeXatLxBI9gBaebbnrfifHhDYfgasaacH8akY=wiFfYdH8Gipec8Eeeu0xXdbba9frFj0=OqFfea0dXdd9vqai=hGuQ8kuc9pgc9s8qqaq=dirpe0xb9q8qiLsFr0=vr0=vr0dc8meaabaqaciaacaGaaeqabaqabeGadaaakeaacqWGZbWCdaqhaaWcbaGaem4AaSMaemiBaWgabaGaey4fIOcaaOGaeyypa0JaemOCai3aa0baaSqaaiabdUgaRjabdYgaSbqaaiabgEHiQaaakmaakaaabaGaemODay3aa0baaSqaaiabdUgaRbqaaiabgEHiQaaakiabdAha2naaDaaaleaacqWGSbaBaeaacqGHxiIkaaaabeaakiaaxMaacaWLjaWaaeWaaeaacqaI0aanaiaawIcacaGLPaaaaaa@4423@

with

rkl∗=(1−λ^1∗)rkl(5)
 MathType@MTEF@5@5@+=feaafiart1ev1aaatCvAUfKttLearuWrP9MDH5MBPbIqV92AaeXatLxBI9gBaebbnrfifHhDYfgasaacH8akY=wiFfYdH8Gipec8Eeeu0xXdbba9frFj0=OqFfea0dXdd9vqai=hGuQ8kuc9pgc9s8qqaq=dirpe0xb9q8qiLsFr0=vr0=vr0dc8meaabaqaciaacaGaaeqabaqabeGadaaakeaacqWGYbGCdaqhaaWcbaGaem4AaSMaemiBaWgabaGaey4fIOcaaOGaeyypa0JaeiikaGIaeGymaeJaeyOeI0ccciGaf83UdWMbaKaadaqhaaWcbaGaeGymaedabaGaey4fIOcaaOGaeiykaKIaemOCai3aaSbaaSqaaiabdUgaRjabdYgaSbqabaGccaWLjaWaaeWaaeaacqaI1aqnaiaawIcacaGLPaaaaaa@41FB@

vk∗=λ^2∗vmedian+(1−λ^2∗)vk     (6)
 MathType@MTEF@5@5@+=feaafiart1ev1aaatCvAUfKttLearuWrP9MDH5MBPbIqV92AaeXatLxBI9gBaebbnrfifHhDYfgasaacH8akY=wiFfYdH8Gipec8Eeeu0xXdbba9frFj0=OqFfea0dXdd9vqai=hGuQ8kuc9pgc9s8qqaq=dirpe0xb9q8qiLsFr0=vr0=vr0dc8meaabaqaciaacaGaaeqabaqabeGadaaakeaacqWG2bGDdaqhaaWcbaGaem4AaSgabaGaey4fIOcaaOGaeyypa0dcciGaf83UdWMbaKaadaqhaaWcbaGaeGOmaidabaGaey4fIOcaaOGaemODay3aaSbaaSqaaiabd2gaTjabdwgaLjabdsgaKjabdMgaPjabdggaHjabd6gaUbqabaGccqGHRaWkcqGGOaakcqaIXaqmcqGHsislcuWF7oaBgaqcamaaDaaaleaacqaIYaGmaeaacqGHxiIkaaGccqGGPaqkcqWG2bGDdaWgaaWcbaGaem4AaSgabeaakiaaxMaacaWLjaWaaeWaaeaacqaI2aGnaiaawIcacaGLPaaaaaa@4E65@

and

λ^1∗=min⁡(1,∑k≠lVar^(rkl)∑k≠lrkl2)     (7)
 MathType@MTEF@5@5@+=feaafiart1ev1aaatCvAUfKttLearuWrP9MDH5MBPbIqV92AaeXatLxBI9gBaebbnrfifHhDYfgasaacH8akY=wiFfYdH8Gipec8Eeeu0xXdbba9frFj0=OqFfea0dXdd9vqai=hGuQ8kuc9pgc9s8qqaq=dirpe0xb9q8qiLsFr0=vr0=vr0dc8meaabaqaciaacaGaaeqabaqabeGadaaakeaaiiGacuWF7oaBgaqcamaaDaaaleaacqaIXaqmaeaacqGHxiIkaaGccqGH9aqpcyGGTbqBcqGGPbqAcqGGUbGBcqGGOaakcqaIXaqmcqGGSaaldaWcaaqaamaaqababaWaaecaaeaaieaacqGFwbGvcqGFHbqycqGFYbGCaiaawkWaaiabcIcaOiabdkhaYnaaBaaaleaacqWGRbWAcqWGSbaBaeqaaOGaeiykaKcaleaacqWGRbWAcqGHGjsUcqWGSbaBaeqaniabggHiLdaakeaadaaeqaqaaiabdkhaYnaaDaaaleaacqWGRbWAcqWGSbaBaeaacqaIYaGmaaaabaGaem4AaSMaeyiyIKRaemiBaWgabeqdcqGHris5aaaakiabcMcaPiaaxMaacaWLjaWaaeWaaeaacqaI3aWnaiaawIcacaGLPaaaaaa@59F7@

λ^2∗=min⁡(1,∑k=1pVar^(vk)∑k=1p(vk−vmedian)2).     (8)
 MathType@MTEF@5@5@+=feaafiart1ev1aaatCvAUfKttLearuWrP9MDH5MBPbIqV92AaeXatLxBI9gBaebbnrfifHhDYfgasaacH8akY=wiFfYdH8Gipec8Eeeu0xXdbba9frFj0=OqFfea0dXdd9vqai=hGuQ8kuc9pgc9s8qqaq=dirpe0xb9q8qiLsFr0=vr0=vr0dc8meaabaqaciaacaGaaeqabaqabeGadaaakeaaiiGacuWF7oaBgaqcamaaDaaaleaacqaIYaGmaeaacqGHxiIkaaGccqGH9aqpcyGGTbqBcqGGPbqAcqGGUbGBcqGGOaakcqaIXaqmcqGGSaaldaWcaaqaamaaqadabaWaaecaaeaaieaacqGFwbGvcqGFHbqycqGFYbGCaiaawkWaaiabcIcaOiabdAha2naaBaaaleaacqWGRbWAaeqaaOGaeiykaKcaleaacqWGRbWAcqGH9aqpcqaIXaqmaeaacqWGWbaCa0GaeyyeIuoaaOqaamaaqadabaGaeiikaGIaemODay3aaSbaaSqaaiabdUgaRbqabaGccqGHsislcqWG2bGDdaWgaaWcbaGaemyBa0MaemyzauMaemizaqMaemyAaKMaemyyaeMaemOBa4gabeaakiabcMcaPmaaCaaaleqabaGaeGOmaidaaaqaaiabdUgaRjabg2da9iabigdaXaqaaiabdchaWbqdcqGHris5aaaakiabcMcaPiabc6caUiaaxMaacaWLjaWaaeWaaeaacqaI4aaoaiaawIcacaGLPaaaaaa@656B@

The particular choice of the shrinkage intensities λ^1∗
 MathType@MTEF@5@5@+=feaafiart1ev1aaatCvAUfKttLearuWrP9MDH5MBPbIqV92AaeXatLxBI9gBaebbnrfifHhDYfgasaacH8akY=wiFfYdH8Gipec8Eeeu0xXdbba9frFj0=OqFfea0dXdd9vqai=hGuQ8kuc9pgc9s8qqaq=dirpe0xb9q8qiLsFr0=vr0=vr0dc8meaabaqaciaacaGaaeqabaqabeGadaaakeaaiiGacuWF7oaBgaqcamaaDaaaleaacqaIXaqmaeaacqGHxiIkaaaaaa@3083@ and λ^2∗
 MathType@MTEF@5@5@+=feaafiart1ev1aaatCvAUfKttLearuWrP9MDH5MBPbIqV92AaeXatLxBI9gBaebbnrfifHhDYfgasaacH8akY=wiFfYdH8Gipec8Eeeu0xXdbba9frFj0=OqFfea0dXdd9vqai=hGuQ8kuc9pgc9s8qqaq=dirpe0xb9q8qiLsFr0=vr0=vr0dc8meaabaqaciaacaGaaeqabaqabeGadaaakeaaiiGacuWF7oaBgaqcamaaDaaaleaacqaIYaGmaeaacqGHxiIkaaaaaa@3085@ is aimed at minimizing the overall mean squared error.

### Shrinkage estimation of VAR coefficients

Small sample shrinkage estimates of VAR regression coefficients may be obtained by appropriately substituting the empirical by the shrinkage covariance. More specifically, we need to proceed as follows:

1. We combine the centered observations ***X***_*p *_and ***X***_*f *_into a joint matrix Φ = [***X***_*p*_***X***_*f*_]. Note that F contains twice as many columns as either ***X***_*p *_or ***X***_*f*_.

2. Next, we consider the (*n *- 1) multiple of the *empirical *covariance matrix, ***S ***= Φ^*T*^Φ, noting that ***S ***contains the two submatrices ***S***_1 _= ***X***_*p *_^*T *^***X***_*p *_and ***S***_2 _= ***X***_*p *_^*T*^***X***_*f*_. This allows to write the OLS estimate of VAR coefficients as B^
 MathType@MTEF@5@5@+=feaafiart1ev1aaatCvAUfKttLearuWrP9MDH5MBPbIqV92AaeXatLxBI9gBaebbnrfifHhDYfgasaacH8akY=wiFfYdH8Gipec8Eeeu0xXdbba9frFj0=OqFfea0dXdd9vqai=hGuQ8kuc9pgc9s8qqaq=dirpe0xb9q8qiLsFr0=vr0=vr0dc8meaabaqaciaacaGaaeqabaqabeGadaaakeaaieWacuWFcbGqgaqcaaaa@2DD1@^OLS ^= (***S***_1_)^-1^***S***_2_.

3. We replace the empirical covariance matrix ***S ***by a shrinkage estimate.

4. From S* we determine the submatrices S1∗
 MathType@MTEF@5@5@+=feaafiart1ev1aaatCvAUfKttLearuWrP9MDH5MBPbIqV92AaeXatLxBI9gBaebbnrfifHhDYfgasaacH8akY=wiFfYdH8Gipec8Eeeu0xXdbba9frFj0=OqFfea0dXdd9vqai=hGuQ8kuc9pgc9s8qqaq=dirpe0xb9q8qiLsFr0=vr0=vr0dc8meaabaqaciaacaGaaeqabaqabeGadaaakeaaieWacqWFtbWudaqhaaWcbaGaeGymaedabaGaey4fIOcaaaaa@2FEF@ and S2∗
 MathType@MTEF@5@5@+=feaafiart1ev1aaatCvAUfKttLearuWrP9MDH5MBPbIqV92AaeXatLxBI9gBaebbnrfifHhDYfgasaacH8akY=wiFfYdH8Gipec8Eeeu0xXdbba9frFj0=OqFfea0dXdd9vqai=hGuQ8kuc9pgc9s8qqaq=dirpe0xb9q8qiLsFr0=vr0=vr0dc8meaabaqaciaacaGaaeqabaqabeGadaaakeaaieWacqWFtbWudaqhaaWcbaGaeGOmaidabaGaey4fIOcaaaaa@2FF1@ which in turn allow to compute the estimates

B^
 MathType@MTEF@5@5@+=feaafiart1ev1aaatCvAUfKttLearuWrP9MDH5MBPbIqV92AaeXatLxBI9gBaebbnrfifHhDYfgasaacH8akY=wiFfYdH8Gipec8Eeeu0xXdbba9frFj0=OqFfea0dXdd9vqai=hGuQ8kuc9pgc9s8qqaq=dirpe0xb9q8qiLsFr0=vr0=vr0dc8meaabaqaciaacaGaaeqabaqabeGadaaakeaaieWacuWFcbGqgaqcaaaa@2DD1@^Shrink ^= (S1∗
 MathType@MTEF@5@5@+=feaafiart1ev1aaatCvAUfKttLearuWrP9MDH5MBPbIqV92AaeXatLxBI9gBaebbnrfifHhDYfgasaacH8akY=wiFfYdH8Gipec8Eeeu0xXdbba9frFj0=OqFfea0dXdd9vqai=hGuQ8kuc9pgc9s8qqaq=dirpe0xb9q8qiLsFr0=vr0=vr0dc8meaabaqaciaacaGaaeqabaqabeGadaaakeaaieWacqWFtbWudaqhaaWcbaGaeGymaedabaGaey4fIOcaaaaa@2FEF@)^-1^S2∗
 MathType@MTEF@5@5@+=feaafiart1ev1aaatCvAUfKttLearuWrP9MDH5MBPbIqV92AaeXatLxBI9gBaebbnrfifHhDYfgasaacH8akY=wiFfYdH8Gipec8Eeeu0xXdbba9frFj0=OqFfea0dXdd9vqai=hGuQ8kuc9pgc9s8qqaq=dirpe0xb9q8qiLsFr0=vr0=vr0dc8meaabaqaciaacaGaaeqabaqabeGadaaakeaaieWacqWFtbWudaqhaaWcbaGaeGOmaidabaGaey4fIOcaaaaa@2FF1@.

By decomposing S* using the SVD or Cholesky algorithm it is possible to reconstruct pseudodata matrices Xf∗
 MathType@MTEF@5@5@+=feaafiart1ev1aaatCvAUfKttLearuWrP9MDH5MBPbIqV92AaeXatLxBI9gBaebbnrfifHhDYfgasaacH8akY=wiFfYdH8Gipec8Eeeu0xXdbba9frFj0=OqFfea0dXdd9vqai=hGuQ8kuc9pgc9s8qqaq=dirpe0xb9q8qiLsFr0=vr0=vr0dc8meaabaqaciaacaGaaeqabaqabeGadaaakeaaieWacqWFybawdaqhaaWcbaGaemOzaygabaGaey4fIOcaaaaa@305E@ and Xp∗
 MathType@MTEF@5@5@+=feaafiart1ev1aaatCvAUfKttLearuWrP9MDH5MBPbIqV92AaeXatLxBI9gBaebbnrfifHhDYfgasaacH8akY=wiFfYdH8Gipec8Eeeu0xXdbba9frFj0=OqFfea0dXdd9vqai=hGuQ8kuc9pgc9s8qqaq=dirpe0xb9q8qiLsFr0=vr0=vr0dc8meaabaqaciaacaGaaeqabaqabeGadaaakeaaieWacqWFybawdaqhaaWcbaGaemiCaahabaGaey4fIOcaaaaa@3072@. The above algorithm may be interpreted as OLS or normal-distribution ML based on these pseudodata.

### VAR network model selection

The network representing potential directed causal influences is given by the non-zero entries in the matrix of VAR coefficient. For an extensive discussion of the meaning and interpretation of the implied Granger (non)-causality we refer to [8].

As B^
 MathType@MTEF@5@5@+=feaafiart1ev1aaatCvAUfKttLearuWrP9MDH5MBPbIqV92AaeXatLxBI9gBaebbnrfifHhDYfgasaacH8akY=wiFfYdH8Gipec8Eeeu0xXdbba9frFj0=OqFfea0dXdd9vqai=hGuQ8kuc9pgc9s8qqaq=dirpe0xb9q8qiLsFr0=vr0=vr0dc8meaabaqaciaacaGaaeqabaqabeGadaaakeaaieWacuWFcbGqgaqcaaaa@2DD1@^Shrink ^is an estimate it is unlikely that any of its components are exactly zero. Therefore, we need to statistically test whether the entries of B^
 MathType@MTEF@5@5@+=feaafiart1ev1aaatCvAUfKttLearuWrP9MDH5MBPbIqV92AaeXatLxBI9gBaebbnrfifHhDYfgasaacH8akY=wiFfYdH8Gipec8Eeeu0xXdbba9frFj0=OqFfea0dXdd9vqai=hGuQ8kuc9pgc9s8qqaq=dirpe0xb9q8qiLsFr0=vr0=vr0dc8meaabaqaciaacaGaaeqabaqabeGadaaakeaaieWacuWFcbGqgaqcaaaa@2DD1@^Shrink ^are vanishing. However, instead of inspecting regression coefficients directly, it is preferably to test the corresponding partial correlation coefficients: this facilitates small-sample testing and additionally allows to accommodate for dependencies among the estimated coefficients [9].

Specifically, consider in the VAR(1) model the multiple regression that connects the first variable *x*_1_(*t *+ 1) at time point *t *+ 1 with all variables *x*_1_(*t*),...,*x*_*p*_(*t*) at the previous time point *t*,

x1(t+1)=c+βk1xk(t)+∑j=1,j≠kpβj1xj(t)+ error.     (9)
 MathType@MTEF@5@5@+=feaafiart1ev1aaatCvAUfKttLearuWrP9MDH5MBPbIqV92AaeXatLxBI9gBaebbnrfifHhDYfgasaacH8akY=wiFfYdH8Gipec8Eeeu0xXdbba9frFj0=OqFfea0dXdd9vqai=hGuQ8kuc9pgc9s8qqaq=dirpe0xb9q8qiLsFr0=vr0=vr0dc8meaabaqaciaacaGaaeqabaqabeGadaaakeaacqWG4baEdaWgaaWcbaGaeGymaedabeaakiabcIcaOiabdsha0jabgUcaRiabigdaXiabcMcaPiabg2da9iabdogaJjabgUcaRGGaciab=j7aInaaDaaaleaacqWGRbWAaeaacqaIXaqmaaGccqWG4baEdaWgaaWcbaGaem4AaSgabeaakiabcIcaOiabdsha0jabcMcaPiabgUcaRmaaqahabaGae8NSdi2aa0baaSqaaiabdQgaQbqaaiabigdaXaaakiabdIha4naaBaaaleaacqWGQbGAaeqaaOGaeiikaGIaemiDaqNaeiykaKIaey4kaSIaaGPaVdWcbaGaemOAaOMaeyypa0JaeGymaeJaeiilaWIaemOAaOMaeyiyIKRaem4AaSgabaGaemiCaahaniabggHiLdacbaGccqGFLbqzcqGFYbGCcqGFYbGCcqGFVbWBcqGFYbGCcqGGUaGlcaWLjaGaaCzcamaabmaabaGaeGyoaKdacaGLOaGaayzkaaaaaa@6781@

If in this equation the roles of *x*_*k*_(*t*) and *x*_1_(*t *+ 1) are reversed,

xk(t)=c+β1kx1(t+1)+∑j=1,j≠kpβ1jxj(t)+ error,     (10)
 MathType@MTEF@5@5@+=feaafiart1ev1aaatCvAUfKttLearuWrP9MDH5MBPbIqV92AaeXatLxBI9gBaebbnrfifHhDYfgasaacH8akY=wiFfYdH8Gipec8Eeeu0xXdbba9frFj0=OqFfea0dXdd9vqai=hGuQ8kuc9pgc9s8qqaq=dirpe0xb9q8qiLsFr0=vr0=vr0dc8meaabaqaciaacaGaaeqabaqabeGadaaakeaacqWG4baEdaWgaaWcbaGaem4AaSgabeaakiabcIcaOiabdsha0jabcMcaPiabg2da9iabdogaJjabgUcaRGGaciab=j7aInaaDaaaleaacqaIXaqmaeaacqWGRbWAaaGccqWG4baEdaWgaaWcbaGaeGymaedabeaakiabcIcaOiabdsha0jabgUcaRiabigdaXiabcMcaPiabgUcaRmaaqahabaGae8NSdi2aa0baaSqaaiabigdaXaqaaiabdQgaQbaakiabdIha4naaBaaaleaacqWGQbGAaeqaaOGaeiikaGIaemiDaqNaeiykaKIaey4kaSIaaGPaVdWcbaGaemOAaOMaeyypa0JaeGymaeJaeiilaWIaemOAaOMaeyiyIKRaem4AaSgabaGaemiCaahaniabggHiLdacbaGccqGFLbqzcqGFYbGCcqGFYbGCcqGFVbWBcqGFYbGCcqGFSaalcaWLjaGaaCzcamaabmaabaGaeGymaeJaeGimaadacaGLOaGaayzkaaaaaa@6857@

the partial correlation between the two variables is the geometric mean of the corresponding regression coefficients, times their sign, i.e. β1kβk1sgn⁡(β1k)
 MathType@MTEF@5@5@+=feaafiart1ev1aaatCvAUfKttLearuWrP9MDH5MBPbIqV92AaeXatLxBI9gBaebbnrfifHhDYfgasaacH8akY=wiFfYdH8Gipec8Eeeu0xXdbba9frFj0=OqFfea0dXdd9vqai=hGuQ8kuc9pgc9s8qqaq=dirpe0xb9q8qiLsFr0=vr0=vr0dc8meaabaqaciaacaGaaeqabaqabeGadaaakeaadaGcaaqaaGGaciab=j7aInaaDaaaleaacqaIXaqmaeaacqWGRbWAaaGccqWFYoGydaqhaaWcbaGaem4AaSgabaGaeGymaedaaaqabaGccyGGZbWCcqGGNbWzcqGGUbGBcqGGOaakcqWFYoGydaqhaaWcbaGaeGymaedabaGaem4AaSgaaOGaeiykaKcaaa@3F0A@[10].

Once the partial correlations in the VAR model are computed, we use the *"local fdr" *approach of [11] to identify significant partial correlations, similar to the network model selection for graphical Gaussian models (GGMs) of [9]. Note that unlike in a GGM the edges in a VAR network are directed.

We point out that recently two papers have appeared describing related strategies for VAR model selection. As in our algorithm the strategies pursued in both [12] and [13] also consist in choosing the VAR network by selecting the appropriate underlying partial correlations. However, the key advantage of our variant of VAR network search is that it is specifically designed to meet small sample requirements, by using shrinkage estimators of regression coefficients and partial correlation, and due to the adaptive nature (i.e. the automatic estimation of the empirical null) of the "local fdr" algorithm [11].

## Results and discussion

### Simulation study

In a comparative simulation study we investigated the power of diverse approaches to recovering the true VAR network. We simulated VAR(1) data of different sample size, with *n *varying between 5 and 200, for 100 randomly generated true networks with 200 edges and *p *= 100 nodes. The 200 nonzero regression coefficients were drawn uniformly from the intervals [-1; -0.2] and [0.2; 1].

In addition to the shrinkage procedure we estimated regression coefficients by ordinary least squares (OLS) and by ridge regression (RR). All these three regression strategies were applied in conjunction with the above VAR model selection based on partial correlations, with a cutoff value for the "local fdr" statistic set at 0.2 – the recommendation of [11]. As a fourth method we employed L1 regression [14] (LASSO) to estimate VAR regression coefficients. Note that in the latter instance there is no need for additional model selection, as the LASSO method combines shrinkage and model selection and automatically sets many regression coefficients identically to zero.

In the simulations we ran OLS only for *n *> 100, as for small sample size the corresponding empirical covariance matrix is singular and consequently the OLS regression is ill-posed. The penalty for the LASSO regression was chosen as in [15]. The regularization parameter in RR was determined by generalized cross validation [16]. Unfortunately, even GCV turned out to be computationally expensive, so that for RR we conducted only 10 repetitions, rather than the 100 considered for the other methods.

**Figure 1:**
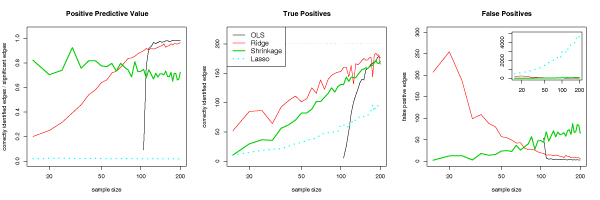
Relative performance of the four investigated methods for learning VAR networks in terms of positive predictive value (true discovery rate) and the number of true and false edges. The thin dotted line in the middle box at 200 corresponds to the true number of edges in the simulated networks.

The results of the simulations are summarized in Figure 1. The left box shows the positive predictive value, or true discovery rate of the four methods. This is the proportion of correctly identified edges in relation to all significant edges. Our proposed shrinkage algorithm is the only method achieving around 80% positive predictive value regardless of the sample size. Note that this is exactly the theoretically expected value, given the specified "local fdr" cutoff of 0.2. In contrast, the RR and LASSO methods perform remarkably poor at small sample size, with much lower true discovery rates. For medium to large sample size the OLS estimation dominates RR, LASSO and the shrinkage approach. This is easily explained by the fact that OLS has no parameters to optimize and that it is asymptotically optimal. However, it is bothering that for both the RR and the OLS approach the false discovery rate appears not to be properly controlled. Finally, for large sample size the Stein-type estimator appears to be prone to overshrinking, which leads to an increase of false positives.

The relative performance of the four approaches to VAR estimation can be further explained by considering the relative amount of true and false positive edges (Figure 1, middle and right box). The shrinkage method generally produces very few false positives. In contrast, the RR and LASSO methods lead to a large number of false edges, especially for small sample size. This is particularly pronounced for the LASSO regression, as can be seen in the differently scaled inlay plot contained in the right box of Figure 1, indicating that the penalty applied in the L1 regression may not be sufficient in this situation. In terms of the number of correctly identified edges the RR and shrinkage approach are the two top performing methods. However, even though RR finds a considerable number of true edges even at very small sample size, this has little impact on its true discovery rate because of the high number of false positives.

In summary, the simulation results suggest to apply for small sample size the James-Stein-type shrinkage procedure, and for *n *>*p *the traditional OLS approach.

### Analysis of a microarray time course data set

For further illustration we applied the VAR shrinkage approach to a real world data example. Specifically, we reanalyzed expression time series resulting from an experiment investigating the impact of the diurnal cycle on the starch metabolism of *Arabidopsis thaliana *[17].

We downloaded the calibrated signal intensities for 22,814 probes and 11 time points for each of the two biological replicates from experiment no. 60 of the NASCArrays repository [18]. After log-transforming the data we filtered out all genes containing missing values and whose maximum signal intensity value was lower than 5 on a log-base 2 scale. Subsequently, we applied the periodicity test of [19] to identify the probes associated with the day-night cycle. As a result, we obtained a subset of 800 genes that we further analyzed with the VAR approach.

We note that a tacit assumption of the VAR model is that time points are equidistant – see Eq. 1. This is not the case for the *Arabidopsis thaliana *data which were measured at 0, 1, 2, 4, 8, 12, 13, 14, 16, 20, and 24 hours after the start of the experiment. However, as the intensity of the biological reactions is likely to be higher at the change points from light to dark periods (time points 0 and 12), one may argue that assuming equidistant measurements is justifiable at least in terms of equal relative reaction rate.

A further implication of the VAR model (and indeed of many other graphical models) is that dependencies among genes are essentially linear. This can easily be checked by inspecting the pairwise scatter plots of the calibrated expression levels. For the 800 considered *Arabidopsis thaliana *genes we verified that the linearity assumption of the VAR model is indeed satisfied.

**Figure 2:**
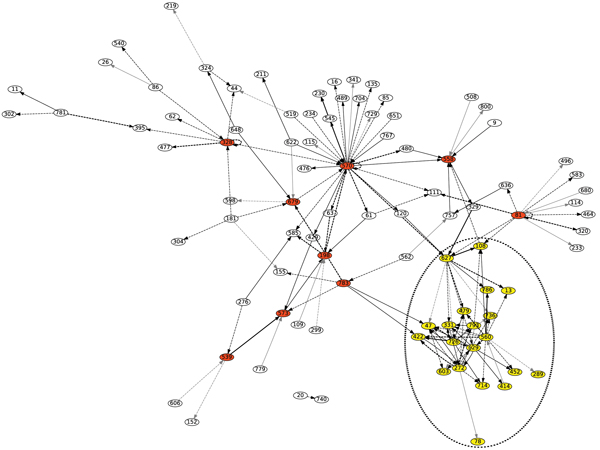
Directed VAR network inferred from the *Arabidopsis thaliana *data. The solid and dotted lines indicate positive and negative regression coefficients, respectively, and the line intensity denotes their strength. For annotation of the nodes see the *Supplementary Information*, Table 1. The color code of the nodes is explained in the main text.

Subsequently, we estimated from the replicate time series of the 800 preselected genes the regularized regression coefficients and the corresponding partial correlations, and identified the significant edges of the VAR causal graph as described above. We found a total number of 7, 381 significant edges connecting 707 nodes. In Figure 2 we show for reasons of clarity only the subnetwork containing the 150 most significant edges, which connect 92 nodes. Note that this graph exhibits a clear "hub" connectivity structure (nodes filled with red color), which is particularly striking for the node 570 but also for nodes 81, 558, 783 and a few other genes (for annotation of the nodes see the Additional File 1).

As the VAR network contains directed edges it is possible to distinguish genes that have mostly outgoing arcs, which could be indicative for key regulatory genes, from those with mostly ingoing arcs. In the graph of Figure 2 node 570, an AP2 transcription factor, and node 81, a gene involved in DNA-directed RNA polymerase, belong to the former category, whereas for instance node 558, a structural constituent of ribosome, seems to be part of the latter. Node 627 is another hub in the VAR network, which according to the annotation of [17] encodes a protein of unknown function. Another interesting aspect of the VAR network is the web of highly connected genes (encircled and colored yellow in the lower right corner of Figure 2) which we hypothesize to constitute some form of a functional module.

**Figure 3:**
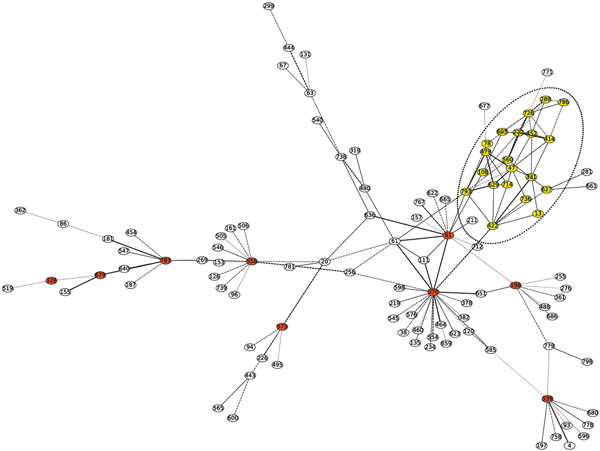
Undirected GGM network inferred from the *Arabidopsis thaliana *data using the algorithm of [7; 9]. The solid and dotted lines indicate positive and negative partial correlation coefficients, respectively, and the line intensity denotes their strength.

Finally, we note that the VAR network visualizes influences of the genes over time, hence a VAR graph can also include directed loops and even genes that act upon themselves. In contrast, the GGM graphs discussed in [7,9] visualize the partial correlation with no time lag involved. For comparison, we display the GGM graph for the *Arabidopsis thaliana *data in Figure 3. As expected, both graphs share the same structure (main hubs and the module of highly connected genes): if one node influences another in the next timepoint with a constant regression coefficient (VAR-model), they also tend to be significantly partially correlated in the same time point (GGM-model). However, using a GGM it is not possible to infer the causal structure of the network.

## Conclusion

We have presented a novel algorithm for learning VAR causal networks. This is based on James-Stein-type shrinkage estimation of covariances between different time points of the conducted experiment, that in turn leads to improved estimates of the VAR regression coefficients. Subsequent VAR model selection is conducted by using "local fdr" multiple testing on the corresponding partial correlations.

We have shown that this approach is well suited for the small sample sizes encountered in genomics. In addition, the approach is computationally very efficient, as no computer intensive sampling or optimization is needed: the inference of the directed network for the *Arabidopsis thaliana *data with 640, 000 potentially directed edges takes about one minute on a standard desktop computer. While we have illustrated the approach by analyzing a microarray expression data set, it is by no means restricted to this kind of data – we expect that our VAR network approach performs equally well for similar high dimensional time series data from metabolomic or proteomic experiments.

The current algorithm employs a fixed "one step ahead" time lag. One strategy to generalization to arbitrary time lags may be to consider functional data – see, e.g., [20,21]. This would have the additional benefit to suitable deal with non-equally spaced measurements, a common characteristic of many biological experiments.

## Authors' contributions

Both authors participated in the development of the methodology and wrote the manuscript. R.O. carried out all analyzes and simulations. All authors approved of the final version of the manuscript.

## Supplementary Material

Additional File 1 This PDF file contains a multi-page table containing the annotation data (probe IDs, gene names, description) for the 92 genes displayed in the VAR network of Figure 2. Click here for file
