# Supplementary material for: Learning causal networks from systems biology time course data: an effective model selection procedure for the vector autoregressive process
Source: BMC Bioinformatics. 2007 May 3;8(Suppl 2):S3. doi: 10.1186/1471-2105-8-S2-S3 (PMC1892072; doi:10.1186/1471-2105-8-S2-S3)
Supplement: Additional File 1 — This PDF file contains a multi-page table containing the annotation data (probe IDs, gene names, description) for the 92 genes displayed in the VAR network of Figure 2. [file 1471-2105-8-S2-S3-S1.pdf]

Additional file 1 - annotation.pdf

This PDF file contains a multi-page table containing the annotation data (probe IDs, gene names, description) for the 92 genes displayed in the VAR network of Figure 2.

Node	Probe ID	Gene Name	Description
9	267432_at	At2g35020-MinT-G	putative UDP-N-acetylglucosamine pyrophosphorylase
11	267383_at	AT2G44360-TAIR-G	unknown protein; expressed protein
13	267341_at	AT2G44200-TAIR-G	unknown protein; expressed protein
16	267123_at	AT2G23560-TAIR-G	catalytic/ hydrolase; hydrolase, alpha/beta fold family protein, similar to ethylene-induced esterase (Citrus sinensis) GI:14279437, polyneuridine aldehyde esterase (Rauvolfia serpentina) GI:6651393; contains Pfam profile PF00561: hydrolase, alpha/beta fold family
20	266995_at	At2g34500-MinT-G	putative cytochrome P450
26	266897_at	AT2G45820-TAIR-G	DNA binding; DNA-binding protein, putative, identical to DNA-binding protein gi—601843—gb—AAA57124 (Arabidopsis thaliana); contains Pfam domain, PF03766: Remorin, N-terminal region; contains Pfam domain, PF03763: Remorin, C-terminal region
44	266314_at	AT2G27040-TAIR-G	AGO4 (ARGONAUTE 4); PAZ domain-containing protein / piwi domain-containing protein, similar to SP—Q9QZ81 Eukaryotic translation initiation factor 2C 2 Rattus norvegicus; contains Pfam profiles PF02171: Piwi domain, PF02170: PAZ domain
47	266247_at	AT2G27660-TAIR-G	unknown protein; DC1 domain-containing protein, contains Pfam profile PF03107: DC1 domain
61	265721_at	AT2G40090-TAIR-G	ATATH9; member of ATH subfamily

62	265695_at	AT2G24490-TAIR-G	nucleic acid binding; replication protein, putative, similar to replication protein A 30kDa (Oryza sativa (japonica cultivar-group)) GI:13516746; contains InterPro entry IPR004365: OB-fold nucleic acid binding domain
63	265674_at	AT2G32190-TAIR-G	unknown protein; expressed protein
78	264986_at	At1g27130-MinT-G	glutathione transferase, putative
81	264924_at	AT1G60620-TAIR-G	ATRPAC43; DNA binding / DNA-directed RNA polymerase; DNA-directed RNA polymerase, putative, identical to RNA polymerase subunit (Arabidopsis thaliana) GI:514324; contains Pfam profile PF01000: RNA polymerase Rpb3/RpoA insert domain
85	264837_at	AT1G03600-TAIR-G	unknown protein; photosystem II family protein, similar to SP:P74367 Synechocystis sp.; similar to ESTs emb—Z27038, gb—AA451546, emb—Z29876, gb—T45359 and gb—R90316
86	264832_at	AT1G03660-TAIR-G	unknown protein; expressed protein, contains 2 predicted transmembrane domains; expression supported by MPSS
108	264179_at	AT1G02180-TAIR-G	unknown protein; ferredoxin-related, similar to Ferredoxin. (SP:O78510) (Cryptomonas phi) Guillardia theta
109	264131_at	At1g79150-MinT-G	hypothetical protein
111	264063_at	At2g27910-MinT-G	unknown protein
114	264038_at	At2g03690-MinT-G	putative ubiquinone biosynthesis protein
115	264004_at	At2g22425-MinT-G	unknown protein
120	263852_at	At2g04450-MinT-G	putative mutT domain protein
135	263489_at	At2g31830-MinT-G	putative inositol polyphosphate 5'-phosphatase
152	263193_at	AT1G36050-STG	unknown protein
155	263047_at	AT2G17630-TAIR-G	phosphoserine transaminase/ transaminase; phosphoserine aminotransferase, putative, similar to Phosphoserine aminotransferase, chloroplast precursor (PSAT) (SP:Q96255) (Arabidopsis thaliana); contains TIGRFAM TIGR01364: phosphoserine aminotransferase; contains Pfam PF00266: aminotransferase, class V

181	262501_at	At1g21690-MinT-G	putative replication factor C subunit
198	262134_at	AT1G77990-TAIR-G	AST56; sulfate transporter; cDNA encoding a sulfate transporter.
211	261810_at	At1g08130-MinT-G	DNA ligase
219	261696_at	AT1G08470-TAIR-G	strictosidine synthase; strictosidine synthase family protein, similar to strictosidine synthase (Rauvolfia serpentina)(SP—P15324)
230	261484_at	AT1G14400-TAIR-G	UBC1 (UBIQUITIN CARRIER PROTEIN 1); ubiquitin conjugating enzyme/ ubiquitin-like activating enzyme; ubiquitin carrier protein (UBC1) mRNA, complete cds
233	261440_at	AT1G28510-TAIR-G	unknown protein; expressed protein
234	261425_at	At1g18880-MinT-G	unknown protein
272	260143_at	AT1G71880-TAIR-G	SUC1; carbohydrate transporter/ sucrose:hydrogen symporter/ sugar porter; member of Sucrose-proton symporter family
276	260099_at	AT1G73180-TAIR-G	unknown protein; eukaryotic translation initiation factor-related, similar to eukaryotic translation initiation factor 2A (GI:21956484) (Homo sapiens); similar to Eukaryotic translation initiation factor 3 subunit 9 (eIF-3 eta) (eIF3 p116) (eIF3 p110) (eIF3b) (Swiss-Prot:P55884) (Homo sapiens)
289	259875_s_at	No gene	
299	259645_at	AT1G69010-TAIR-G	DNA binding / transcription factor; basic helix-loop-helix (bHLH) family protein, contains Pfam profile: PF00010 helix-loop-helix DNA-binding domain
302	259488_at	AT1G15780-TAIR-G	unknown protein; expressed protein
304	259406_at	At1g17690-MinT-G	unknown protein
320	259111_at	At3g05520-MinT-G	alpha subunit of F-actin capping protein
324	259069_at	At3g11710-MinT-G	lysyl-tRNA synthetase
328	258871_at	AT3G03060-TAIR-G	ATP binding / ATPase/ nucleoside-triphosphatase/ nucleotide binding; AAA-type ATPase family protein, contains a ATP/GTP-binding site motif A (P-loop), PROSITE:PS00017

329	258781_at	AT3G11740-TAIR-G	unknown protein; expressed protein, similar to expressed protein [Arabidopsis thaliana] (TAIR:At5g01750.2); similar to hypothetical protein [Oryza sativa (japonica cultivar-group)] (GB:XP_470102.1); contains InterPro domain Protein of unknown function DUF567 (InterPro:IPR007612)
331	258764_at	AT3G10720-TAIR-G	enzyme inhibitor/ pectinesterase; pectinesterase, putative, contains similarity to pectinesterase from Vitis vinifera GI:15081598, Prunus persica SP—Q43062; contains Pfam profile PF01095 pectinesterase
341	258432_at	At3g16570-MinT-G	unknown protein
395	256626_at	AT3G20015-TAIR-G	pepsin A; similar to aspartyl protease family protein [Arabidopsis thaliana] (TAIR:At3g18490.1); similar to putative aspartic proteinase nepenthesin I [Oryza sativa (japonica cultivar-group)] (GB:NP_909181.1); contains InterPro domain Aspartic protease A1, pepsin (InterPro:IPR001461)
414	256169_at	At1g51800-MinT-G	unknown protein
422	255844_at	AT2G33580-TAIR-G	kinase; protein kinase family protein / peptidoglycan-binding LysM domain-containing protein, protein kinase (Arabidopsis thaliana) GI:2852449; contains Pfam profiles PF01476: LysM domain, PF00069: Protein kinase domain
429	255645_at	AT4G00880-TAIR-G	unknown protein; auxin-responsive family protein, similar to small auxin up RNA (GI:546362) Arabidopsis thaliana
452	254785_at	At4g12730-MinT-G	fasciclin-like arabinogalactan protein FLA2
464	254515_at	At4g20270-MinT-G	CLV1 receptor kinase like protein
476	254239_at	AT4G23400-TAIR-G	PIP1;5/PIP1D; water channel; major intrinsic family protein / MIP family protein, contains Pfam profile: MIP PF00230

477	254233_at	AT4G23800-TAIR-G	transcription factor; high mobility group (HMG1/2) family protein, similar to HMG2B (Homo sapiens) GI:32335; contains Pfam profile PF00505: HMG (high mobility group) box
479	254211_at	AT4G23570-TAIR-G	SGT1A; Closely related to SGT1B, may function in SCF(TIR1) mediated protein degradation
480	254162_at	At4g24440-MinT-G	transcription factor IIA small subunit
489	253927_at	AT4G26710-TAIR-G	hydrogen-transporting ATP synthase, rotational mechanism / hydrogen-transporting ATPase, rotational mechanism; ATP synthase subunit H family protein, contains similarity to Swiss-Prot:O15342 Vacuolar ATP synthase subunit H (V-ATPase H subunit) (Vacuolar proton pump H subunit) (V-ATPase M9.2 subunit) (V-ATPase 9.2 kDa membrane accessory protein) (Homo sapiens)
496	253708_at	At4g29210-MinT-G	gamma-glutamyltransferase-like protein
508	253523_at	AT4G31340-TAIR-G	unknown protein; similar to DNA repair ATPase-related [Arabidopsis thaliana] (TAIR:At2g24420.2); similar to DNA repair ATPase-related [Arabidopsis thaliana] (TAIR:At2g24420.1); similar to unknown [Oryza sativa (japonica cultivar-group)] (GB:AAO72581.1)
519	253202_at	AT4G34555-TAIR-G	structural constituent of ribosome; 40S ribosomal protein S25, putative
539	252427_at	AT3G47640-TAIR-G	DNA binding / transcription factor; basic helix-loop-helix (bHLH) family protein, contains Pfam profile: PF00010 helix-loop-helix DNA-binding domain
540	252420_at	At3g47530-MinT-G	putative protein
545	252098_at	At3g51330-MinT-G	predicted GPI-anchored protein
558	251834_at	AT3G55170-TAIR-G	structural constituent of ribosome; 60S ribosomal protein L35 (RPL35C), various ribosomal L35 proteins
560	251786_at	At3g55270-MinT-G	MAP kinase phosphatase (MKP1)

562	251768_at	AT3G55940-TAIR-G	phosphoinositide phospholipase C/ phospholipase C; phosphoinositide-specific phospholipase C, putative, similar to phosphoinositide specific phospholipase C GI:857374 from (Arabidopsis thaliana)
570	251598_at	At3g57600-MinT-G	AP2 transcription factor - like protein
573	251483_at	At3g59650-MinT-G	unknown protein
583	251227_at	At3g62700-MinT-G	ABC transporter-like protein
585	251123_at	AT5G01030-TAIR-G	unknown protein; expressed protein
598	250661_at	At5g07030-MinT-G	nucleoid DNA-binding-like protein
603	250520_at	At5g08470-MinT-G	putative protein
606	250433_at	AT5G10400-TAIR-G	DNA binding; histone H3, identical to several histone H3 proteins, including Zea mays SP—P05203, Medicago sativa GI:166384, Encephalartos altensteinii SP—P08903, Pisum sativum SP—P02300; contains Pfam profile PF00125 Core histone H2A/H2B/H3/H4
622	249997_at	AT5G18620-TAIR-G	ATP binding / ATP-dependent helicase/ DNA binding / DNA-dependent ATPase/ helicase/ nucleic acid binding; DNA-dependent ATPase, putative, similar to DNA-dependent ATPase SNF2H (Mus musculus) GI:14028669; contains Pfam profiles PF00271: Helicase conserved C-terminal domain, PF00176: SNF2 family N-terminal domain, PF00249: Myb-like DNA-binding domain
627	249817_at	AT5G23820-TAIR-G	unknown protein; MD-2-related lipid recognition domain-containing protein / ML domain-containing protein, contains Pfam profile PF02221: ML domain
629	249777_at	AT5G24210-TAIR-G	triacylglycerol lipase; lipase class 3 family protein, contains Pfam profile PF01764: Lipase
636	249569_at	At5g38070-MinT-G	putative protein
648	249315_at	At5g41190-MinT-G	unknown protein
651	249211_at	At5g42680-MinT-G	putative protein
679	248295_at	At5g53070-MinT-G	unknown protein (At5g53070)

680	248248_at	AT5G53120-TAIR-G	SPDS3 (SPERMIDINE SYNTHASE 3); encodes a novel spermine synthase and is a paralog of previously characterized spermidine synthases, SPDS1 and SPDS2. SPDS3 forms heterodimers with SDPS2, which in turn forms heterodimers with SDPS1 in vivo. The gene does not complement speDelta3 deficiency of spermidine synthase in yeast but DOES complement speDelta4 deficiency.
704	247791_at	AT5G58710-TAIR-G	ROC7; peptidyl-prolyl cis-trans isomerase; cyclophilin (ROC7) mRNA, complete cds
714	247554_at	AT5G61010-TAIR-G	protein binding; similar to exocyst subunit EXO70 family protein [Arabidopsis thaliana] (TAIR:At3g29400.1); similar to putative leucine zipper-containing protein [Oryza sativa (japonica cultivar-group)] (GB:XP_465879.1); contains InterPro domain Exo70 exocyst complex subunit (InterPro:IPR004140)
726	247097_at	AT5G66460-TAIR-G	hydrolase, hydrolyzing O-glycosyl compounds; (1-4)-beta-mannan endohydrolase, putative, similar to (1-4)-beta-mannan endohydrolase (Coffea arabica) GI:10178872; contains Pfam profile PF00150: Cellulase (glycosyl hydrolase family 5)
729	247055_at	At5g66740-MinT-G	putative protein
736	246976_s_at	At5g24810-MinT-G	unknown protein
740	246837_at	At5g26670-MinT-G	pectin acetylesterase precursor - like protein
757	246421_at	At5g16880-MinT-G	TOM (target of myb1) -like protein
767	246043_at	AT5G19380-TAIR-G	unknown protein; expressed protein
779	245619_at	AT4G13990-TAIR-G	catalytic; exostosin family protein, contains Pfam profile: PF03016 exostosin family
781	245435_at	At4g17130-MinT-G	hypothetical protein

783	245404_at	AT4G17610-TAIR-G	RNA binding / RNA methyltransferase; tRNA/rRNA methyltransferase (SpoU) family protein, similar to TAR RNA loop binding protein (Homo sapiens) GI:1184692; contains Pfam profile PF00588: SpoU rRNA Methylase (RNA methyltransferase, TrmH) family
786	245347_at	AT4G14890-TAIR-G	electron carrier/ electron transporter/ iron ion binding; ferredoxin family protein, similar to SP—P00252 Ferredoxin I from Nostoc muscorum, SP—P00248 Ferredoxin from Mastigocladus laminosus, SP—P00244 Ferredoxin I from Aphanizomenon flosaquae; contains Pfam profile PF00111 2Fe-2S iron-sulfur cluster binding domain
793	245218_s_at	No gene	
800	244996_at	ATCG00160-TAIR-G	RPS2 (RIBOSOMAL PROTEIN S2); Chloroplast ribosomal protein S2
